# A Rare Case of Widely Disseminated Syphilis

**DOI:** 10.2340/actadv.v104.27983

**Published:** 2024-05-30

**Authors:** Agnieszka SLYK, Maria HEDMAN, Arne WIKSTRÖM

**Affiliations:** Department of Dermatology and Venereology, Karolinska University Hospital, Eugeniavägen 3, Solna, SE-171 76 Stockholm, Sweden

Syphilis is an infection caused by the bacterium *Treponema pallidum,* primarily transmitted through sexual contact, with an estimated 6.3 million cases annually worldwide (1). In 25% of infected patients, syphilis reaches a secondary stage through dissemination, occurring 4–10 weeks post exposure (2).

The “classical triad” manifests as lymphadenopathy, enanthema, and symmetric maculopapular exanthema of the trunk and extremities with palmoplantar involvement (3). *T. Pallidum* can infect any organ system and may lead to neurosyphilis in up to 30% of untreated patients (4, 5).

Syphilitic hepatitis has been described in case reports. Published cases illustrate hepatitis with elevated ALP, normal or slightly elevated transaminases and viral hepatitis-like findings, resolving upon antibiotic treatment (6–8).

## CASE REPORT

A previously healthy 46-year-old woman presented to a gynecologist in Sweden, in September 2022. She complained about vaginal breakthrough bleeding and a foul-smelling discharge. Her sole medication was an oral anti-contraceptive, with no intake of over-the-counter (OTC) medicines. Bacterial vaginosis (BV) was suspected and she received intravaginal clindamycin ovules á 100 mg nightly for 7 days.

**Fig. 1:**
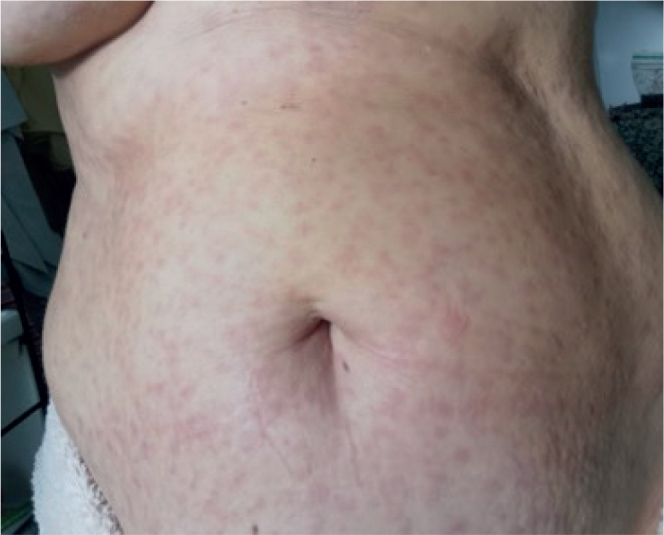
Cutaneous manifestations of lues on patients’ abdomen.

Few days following the BV treatment, she developed a nonpruritic maculopapular exanthema around the areolas, progressing to the trunk and extremities. Face and palmoplantar regions were spared (**Fig. 1**). In October, an exanthematous drug reaction was suspected by a dermatologist in Poland. Prednisolone 40 mg daily for 1,5 weeks and topical steroids was prescribed. Nuchal pain, dysphagia, otalgia and lymphadenopathy followed. A dry cough, night sweats and bilateral hearing loss debuted. In one month, she lost 4 kg of weight.

In November 2022 she was admitted to the dermatology department, at Karolinska University Hospital in Stockholm, Sweden. A drug reaction with eosinophilia and systemic symptoms (DRESS) was suspected.

Whilst her general condition deteriorated, the exanthema partially faded and became pruritic. Her vital parameters were stable. Multiple symptoms developed, such as pharyngitis, otalgia, bilateral wrist arthralgia, left-sided swelling of the neck, and axillary discomfort. Neurological examination revealed blurry vision, hoarseness and phalangeal numbness. Blood analyses showed progressing systemic inflammation and hepatitis (Table SI).

A CT scan from head to pelvis with contrast was performed, showing clogged middle ears, mastoid cells, and sinuses. The liver had an ordinary structure, also confirmed by a following ultrasonography. Lymphadenopathy measuring 9–10 mm was found in the axillae and neck.

Physical examination showed bilateral serous media otitis. A fine needle biopsy from a lymph node in the nuchal region was inconclusive. Tests for autoimmune hepatitis were negative.

In November 2022, microbiological investigation and extended sexually transmitted infections (STIs) screening revealed a positive syphilis serology, with serum IgM of 22,7, serum IgG+IgM of 40,6, serum TPPA >40960, and serum VDRL of 64. She tested negative for Chlamydia trachomatis, Neisseria Gonorrhoeae, Mycoplasma genitalium. Viral serology was negative for Hepatitis A, B, C, Cytomegalovirus (CMV), Parvovirus B19, Human herpes virus (HHV) 6A, 6B and Epstein Barr virus (EBV). Negative PCR for COVID-19, Influensa A, B, Respiratory Syncytial virus (RS) and Mycoplasma pneumoniae, among other pathogens. *E. coli* was found in the urine, for which she received a 3-day course of pivmecillinam.

Routine histopathology, including immunohistochemical staining, confirmed spirochete infection both in the epidermis and in the perivasal adnexal parts of the inflamed dermis.

Cerebrospinal fluid (CSF) analysis demonstrated abnormal cell counts (Table SI), other analyses of neuroinflammation, such as albumin, lactate and glucose, were missing data. She received treatment for neurosyphilis with intravenous penicillin G 3 gram x 4 daily, for 10 days. At day 10, all her symptoms except for nuchal swelling had resolved. Treatment continued with intramuscular injections of benzathine penicillin G 2.4 million units once weekly for 3 weeks. Two months later, she was fully recovered and her blood tests returned to normal (Table SI).

## DISCUSSION

The patient had neurological symptoms, and an elevated leukocyte count upon CSF analysis. All her symptoms resolved after treatment sufficient for neurosyphilis. Later on, treponemal and nontreponemal tests in the CSF turned out negative, and the TPPA serum/liquor ratio could neither confirm neurosyphilis according to Swedish guidelines (9). The investigation showed a widely disseminated, secondary stage syphilitic infection.

Contact tracing revealed that she was likely infected with syphilis in Poland, five months prior to the debut of her symptoms. To evaluate if she had been exposed to *T. pallidum* before, a previous serological specimen for comparison is valuable. She tested negative for syphilis > 20 years ago, during routine pregnancy screening.

The incidence of syphilis in Sweden and Poland is similar, 4,2 and 4,3/100 000 respectively, compared to 7,4/100 000 in Europe and 14,3/100 000 in USA as of 2019. An increase has been seen over the past 10 years in both Europe and USA(10, 11). In Europe, heterosexual females comprise only 10% of the total amount of syphilis infections (10). The patient had been seeking healthcare on multiple occasions without the taking of sexual history, which is likely one of the reasons for the delayed diagnosis. This emphasizes that history taking, including sexual history and travels abroad, is important as both the incidence and knowledge of syphilis varies between countries.

Corticosteroids have a dose dependent immune suppressive effect. It is known that they can worsen the clinical outcome for certain infections (12). The patient was prescribed prednisolone 0,5 mg/kg daily for 1,5 weeks, a dose efficient to treat autoimmune diseases, for instance bullous pemphigoid (13). It raises the suspicion that the corticosteroid immune suppressive effect, aided *T. pallidum* to spread more widely than usually seen. This is supported by the patient’s clinical decline during the prednisolone course. Patients suffering from other immune suppressed states, such as infection with HIV, are also known to have a more aggressive presentation of secondary syphilis (14).

Syphilitic hepatitis (SH) is rare. To our knowledge, there is no epidemiological data of its occurrence. According to a systematic literature review, SH typically features hepatosplenomegaly, high levels of GT/ALP, and mild elevation in ALT/AST (15). The patient had an ordinary liver structure, with high levels of ALT, GT and ALP. She reported a consumption of 2–3 units of alcohol weekly and displayed a very low Phosphatidylethanol (PEth) value, excluding alcoholic liver disease. Alternative causes to her hepatitis other than SH, were ruled out through laboratory investigations. Thus, this patient presents with a unique finding of isolated SH, unlike other case reports that have frequently described SH with concomitant liver disease or HIV (6–8, 15).

To conclude, the infection was widely spread with unusual features, such as hepatitis, neurological symptoms and upper respiratory tract involvement. A drastic improvement in clinical status and laboratory markers was seen after 10 days of treatment. Differential diagnoses such as DRESS, were eventually concluded as unlikely, and her symptomatology could not be well explained by another condition than syphilis.

This is a rare presentation of syphilis. It is important to evaluate cutaneous secondary syphilis, due to its unpredictable dissemination to other organs, and mimicry of different diseases. Clinicians should be aware of syphilitic manifestations as a differential diagnosis of any systemic disorder expressed in the dermis.

## Supplementary Material

A Rare Case of Widely Disseminated Syphilis
