# Supplementary material for: A Rare Case of Widely Disseminated Syphilis
Source: Acta Derm Venereol. 2024 May 30;104:27983. doi: 10.2340/actadv.v104.27983 (PMC11161808; doi:10.2340/actadv.v104.27983)
Supplement: A Rare Case of Widely Disseminated Syphilis [file ActaDV-104-27983-s1.pdf]

Supplementary material has been published as submitted. It has not been copyedited, typeset or checked for scientific content by Acta Dermato-Venereologica

Table SI. Blood analyses before treatment and at 10, 60 days after initiated treatment

Day	Inflammation (blood)						Coagulation		Other
	CRP	SR	Prokalcitonin	Leukocytes	Eosinophils		INR		PEth 16:0/ 18:1
(RV)	(<3)	(<20)	(<0.5)	(3.5-8.8)	(0.0-0.5)		(<1.3)		(<0.05)
0	32	104	0.24	9.8	0.2		1		0.08
10	1	62	x	5.7	0.2		1		
60	x	6	x	5.3	x		x		
	Liver-enzymes (blood)						Autoimmune hepatitis		
	ALP	GT	Bilirubin	ALAT	ASAT	Albumin	ANA	mtDNA	SMA, LKM
(RV)	(0.7-1.9)	(1.3)	(<26)	(<0.76)	(<0.61)	(36-45)	(<1)	(<4)	(neg)
0	12.2	8.7	23	8.57	4.33	26	<0.1	1.5	neg
10	2.9	1.8	3	0.58	0.43	26	x	x	x
60	1	0.25	x	0.3	0.34	40	x	x	x
	Further hematology (blood)								
	Hemoglobin	MCV	MCH	Leukocytes	Trombocytes	LD	Iron	Ferritin	Transferrin
(RV)	(117-153)	(82-98)	(27-33)	(3.5-8.8)	(165-387)	(3.5)	(9-34)	(13-150)	(1.90-3.30)
0	115	86	28	9.8	435	5.3	14	190	1.89
10	111	88	29	5.7	500	x	x	x	x
60	132	90	28	5.3	337	x	x	x	x
	Cerebrospinal fluid (CSF)								
	Clarity	Colour	Leukocytes total	Leukocytes poly	Leukocytes mono	Erythrocytes			
(RV)	(clear)	(colourless)	(0-5)	(<1)	(0-5)	(<1)			
0	Clear	Colourless	26	4	22	5			

Analyses at "day 0" were gathered at days -6 to 0, before initiated antibiotic treatment. Day 10 is the last day of treatment with IV penicillin G.
RV: reference value.
